# Comparative Analysis of Clinical Parameters and Sputum Biomarkers in Establishing the Relevance of Filamentous Fungi in Cystic Fibrosis

**DOI:** 10.3389/fcimb.2020.605241

**Published:** 2021-01-22

**Authors:** Deepa Patel, Keith Chester Dacanay, Catherine H. Pashley, Erol A. Gaillard

**Affiliations:** ^1^ Department of Respiratory Sciences, University of Leicester, Leicester, United Kingdom; ^2^ Paediatric Respiratory Department, University Hospitals of Leicester NHS Trust, Leicester, United Kingdom; ^3^ Institute for Lung Health, NIHR Respiratory Biomedical Research Center, Leicester, United Kingdom

**Keywords:** biomarkers, inflammation, galactomannan, TNF-R2, IL-8, neutrophil, fungi, children

## Abstract

**Background:**

The relationship between fungal culture (FC) positivity and airway inflammation in CF is largely unknown. Identifying the clinical significance of filamentous fungi in CF using both clinical parameters and biomarkers may change our antimicrobial therapeutic strategies.

**Objectives:**

To investigate the clinical characteristics and airway biomarker profile in relation to the detection of filamentous fungi in respiratory samples obtained from CF patients.

**Methods:**

A prospective cohort study over 24 months, including children and adults with CF. Participants provided sputum and/or bronchoalveolar lavage samples, which underwent processing for bacterial and fungal culture, leukocyte differential cell count and biomarker analysis for neutrophil elastase (NE), interleukin-8 (IL-8), galactomannan and tumor necrosis factor receptor type 2 (TNF-R2). We performed FC using neat sputum plugs, an approach shown to be more sensitive compared to routine laboratory testing.

**Results:**

Sixty-one patients provided 76 respiratory samples (72 sputum and 4 BAL). Median age was 17 years (range 6 months–59 years). FC positivity was noted in 49% of the cohort. FC positivity was greater during pulmonary exacerbation compared to the stable state (67 versus 50%). Participants aged 5–30 years had a lower FEV1 within the FC positive group. A significant association between FC positivity and non-tuberculosis mycobacterial (NTM) culture was observed on non-parametric testing (p = 0.022) and regression analysis (p = 0.007). Exposure to indoor mold was a predictor for FC positivity (p = 0.047). There was a trend towards increased lung clearance index (LCI), bronchiectasis and intravenous antibiotic use in the FC positive group. There was no significant difference in biomarkers between FC positive and negative patients.

**Conclusion:**

*Aspergillus. fumigatus* is the commonest filamentous fungi cultured from CF airways. We found no difference in the airway biomarker profile between FC positive and negative patients. The role of galactomannan and TNFR2 as fungal specific biomarkers in CF remains uncertain. FC positivity is associated with a lower FEV_1_ in younger patients, a lower LCI, NTM positivity, bronchiectasis, and intravenous antibiotic exposure. Larger trials are needed to determine the role of galactomannan and TNF-R2 as potential fungal biomarkers in CF.

## Introduction

Cystic fibrosis (CF) lung disease involves impaired mucociliary clearance leading to pulmonary inflammation, infection, and a more rapidly declining lung function. The CF airway milieu contains higher levels of pro-inflammatory cytokines in comparison to healthy controls (Sagel, 2009). Whilst the association between pathogenic bacteria and airway inflammation, infection and disease progression is well established (Hauser et al., 2011), less is known about the role of fungi in the CF airways and their impact on airway inflammation and lung disease progression. Allergic bronchopulmonary aspergillosis (ABPA) is a well-established complication in CF associated with sensitization to *Aspergillus fumigatus* (Thia and Balfour Lynn, 2009). In the absence of ABPA, it is not clear whether the detection of fungi in the CF airway in asymptomatic patients reflects simple colonization (innocent bystander effect) or infection contributing to increased airway inflammation (Liu et al., 2013; Chotirmall and McElvaney, 2014).

*A. fumigatus* is the commonest filamentous fungus isolated from CF airways (Pihet et al., 2009; Sudfeld et al., 2009). Improved mycological methods enable the detection of other fungi, including *Aspergillus terreus, Scedosporium apiospermum* complex, *Exophiala dermatitidis*, and *Penicillium* species (Borman et al., 2010).

The clinical significance of *A. fumigatus* colonization in CF is uncertain. Whilst Saunders et al. (2016) report a steeper decline in Forced Expiratory Volume in 1 second (FEV1) in their fungal culture positive pediatric CF cohort, studies by Baxter et al. (2013b) and De Vrankrijker et al. (2011) found no association between *A. fumigatus* airway colonization and lung function. Other studies have reported greater risk of pulmonary exacerbations requiring hospitalization (Amin, 2010) and higher rates of bronchiectasis (McMahon et al., 2012).

The lack of standardization in processing respiratory samples for fungal culture, differing fungal recovery rates between standard culture methods and the insensitivity of certain fungal culture methods (Chen et al., 2017) is likely to contribute to these contradictory findings. The use of modified culture methods using higher amounts of sputum inoculum have shown increased sensitivity in the detection of fungi (Pashley et al., 2012; Fraczek et al., 2014).

Exploring the relationship between FC positivity and inflammatory airway biomarkers may help in clarifying the clinical significance of fungi. Attempts at profiling the inflammatory cytokine profile in conjunction with clinical and microbiological profiles in CF is limited to a few well-established biomarkers (Frayman et al., 2017) such as neutrophil elastase (NE) and interleukin-8 (IL-8) found in higher concentrations in the CF airway (Frayman et al., 2017). There is a need to identify biomarkers that are specific for pulmonary infections including fungal infection. There is emerging evidence for the novel biomarkers galactomannan and tumor necrosis factor receptor 2 (TNF-R2) for monitoring non-invasive fungal lung disease in CF and asthma (Baxter et al., 2013a; Ghebre et al., 2017)

Neutrophil-driven airway inflammatory responses play a central role in CF. Activation of the innate immune system following pathogenic exposure results in phagocytosis, release of NE and cytokines (Hartl et al., 2012; Lammertyn et al., 2017). IL-1β and TNF-α facilitate recruitment of immune cells to the site of inflammation. Recruitment cytokines such as IL-8 have high neutrophil attracting capacity (Strieter, 2002), further exacerbating the inflammatory process and resulting in large amounts of neutrophilic intracellular protease and oxidant being released. Activation cytokines (interferon-γ, IL-2, IL-12, and TNF-α) augments the T-helper cell response.

Inhalation of fungal spores may result in germination and hyphal growth within the bronchioles (Chotirmall and McElvaney, 2014). Fungi may subsequently colonize, infect, or induce a spectrum of hypersensitivity reactions within the airways. Galactomannan, a fungal cell wall component, is released during hyphal tissue invasion by *Aspergillus* species and can be detected in body fluids (Mennink-Kersten et al., 2004). Current literature focusses on the role of galactomannan in invasive pulmonary aspergillosis, but only a few studies to date have aimed to evaluate its role in non-invasive fungal lung disease (Nguyen et al., 2007; Baxter et al., 2013a).

Differentiating between filamentous fungi as innocent bystanders and fungal infection, using a combination of clinical parameters and airway biomarkers, may inform antimicrobial strategies. This is particularly important given emerging evidence that CF transmembrane conductance regulator (CFTR) modulators may not alter susceptibility to *A. fumigatus* (Fritsch et al., 2019). Consequently the role of fungi in CF is likely to present an ongoing clinical challenge, whilst at the same time limiting our therapeutic options due to complex drug interactions (Tracy and Moss, 2018).

We report a prospective observational cohort study. Our principal aim was to describe the clinical characteristics and airway inflammatory biomarker profile (NE, IL-8, TNF-R2 and galactomannan) in filamentous fungi culture-positive CF patients. We hypothesized that fungal culture positivity in CF is associated with worse lung function and increased airway inflammation.

## Materials and Methods

### Setting and Study Population

Children and adults attending tertiary CF centers at the University Hospitals of Leicester NHS trust, UK, were eligible for enrolment if they had a well-documented diagnosis of CF based on the presence of two disease-causing CFTR mutations and/or a sweat chloride ≥60 mmol/L. Exclusion criteria included CF transplant patients. Participants were approached during routine clinical encounters inclusive of inpatient admissions and outpatient visits. Inclusion criteria for this study included having sputum and/or bronchoalveolar (BAL) samples collected. All respiratory samples were obtained when clinically indicated. Samples during the stable state are obtained at all routine clinic visits on a two-monthly basis or as part of their annual comprehensive review process. Further respiratory samples were obtained during periods of exacerbation as defined by Fuch’s criteria (Fuchs et al., 1994). BAL sampling was undertaken when there was no clinical response to a 4–6-week course of broad spectrum oral antibiotics without a causative pathogen on respiratory samples.

Written informed consent was obtained from study participants ≥17 years of age. For participants <17 years of age written informed consent was obtained from parents or caregivers. Pediatric patients older than 7 years old also provided written assent. This study was approved for review and anonymized use of patient clinical data as part of the Leicester longitudinal study of respiratory infections and microbiomics in CF by the East Midlands Research Ethics Committee, reference number 12/WM/0285.

### Study Design

Prospective cohort study, including pediatric and adult CF patients, from September 2016 to August 2018. The study protocol included a baseline visit which occurred at a routine clinical encounter during a non-exacerbation period. We obtained paired exacerbation and stable respiratory samples for all study participants who experienced a pulmonary exacerbation during the study period.

### Sputum and Data Collection

Pediatric study participants aged five years and over underwent sputum induction (Paggiaro et al., 2002; Ahmed et al., 2019) lung function and serological testing as part of their annual review process during a period of stable disease. Sputum was induced with hypertonic saline in our purpose built negative pressure room dedicated to children. If an insufficient sputum sample was obtained during sputum induction, spontaneously expectorated sputum samples and/or BAL samples were collected according to standardized procedures (Miller et al., 2005). BAL samples were obtained in theatre under anesthesia as recommended by the ERS task force (De Blic et al., 2000). Adult patients do not routinely undergo sputum induction and therefore spontaneously expectorated sputum was used during a stable clinical state.

Electronic medical records were used to obtain study data including: patient demographics, CFTR genotype, medications, lung function, serological, clinical microbiology, and radiology results (bronchiectasis on CT chest). Pediatric patients also underwent a multiple nitrogen washout test (Lum et al., 2007; Davies et al., 2008; Nuttall et al., 2019) as part of their annual review process. We assessed the respiratory status (stable versus exacerbation) of all CF patients at the time of sputum collection.

### Respiratory Sample Processing and Storage

For the detection of filamentous fungi, a sensitive approach was used whereby neat sputum plugs were inoculated onto potato dextrose agar plates containing 16 µl/ml chloramphenicol, 4 µl/ml gentamicin, and 5 µl/ml fluconazole (PGCF) (Pashley et al., 2012). Samples were incubated at 37°C for up to seven days. We identified fungi based on macroscopic and microscopic features (Greer et al., 1997). For bacterial cultures the British National Standard method for investigating sputum was used in accordance with the UK CF Trust Microbiology Standards Working Group recommendations (Public Health England, 2019). Sputum was homogenised with 0.1% dithiothreitol (DTT) and 10 µl of this mixture was diluted in 5 ml distilled water. A sterile loop was used to simultaneously inoculate 1 µl onto the following culture media: (1) mannitol salt/chromogenic agar, (2) cystine-lactose-electrolyte-deficient (CLED) agar, (3) *Burkholderia cepacia* selective agar, and (4) *Mycobacterium species* selective agar at 35–37°C. All samples were incubated for 48 h to five days, except for *Mycobacterium* selective culture, which requires incubation for up to eight weeks.

Supernatant from sputum and/or BAL samples were obtained following homogenisation with 0.1% DTT, followed by vortexing, filtering and a centrifugation step as per our local SOP (Bhatt, 2016).

### Cohort Characterization

Participants were categorized into the following groups; A) fungal culture positive and B) fungal culture negative based on absence of filamentous fungi on culture. Further sub-group analysis included paired samples obtained during pulmonary exacerbation and a clinically stable state. Pulmonary exacerbation was defined by Fuch’s criteria and stable disease included the absence of Fuch’s criteria (Fuchs et al., 1994). Colonization with a known CF pathogen was defined as ≥50% respiratory samples positive for the same bacteria on routine culture within a 12-month period (Liu et al., 2013).

Participants were deemed to have allergic bronchopulmonary aspergillosis (ABPA) (Barton et al., 2008) if they had suggestive clinical features including; a total serum IgE >500 kU/L and a raised specific IgE to *A. fumigatus* of >0.35 kUA/L (Thia and Balfour Lynn, 2009).

### Sputum Cell Differentials

Cytoslides were prepared by fix air-drying in 100% methanol for five minutes followed by staining using RapiDiff II (Bios Europe) staining kit according to protocol (Parker, 2018). The cytoslides were used to determine the leukocyte differential cell count (Brightling et al., 2000).

### ELISA Biomarker Assays

Inflammatory cytokine profiles were measured in sputum supernatants; We estimated NE using the PMN Human Elastase ELISA kit (Invitrogen™), IL-8 using the Human IL-8 ELISA Kit (ThermoFisher Scientific), galactomannan using the Platelia™ Enzyme Immunoassay *Aspergillus* antigen^©^ (Bio-Rad) kit and TNF-R2 using the Human Tumor Necrosis Factor Receptor II (soluble TNF-R2) ELISA kit (Invitrogen ™). All ELISA assays were performed in accordance with individual manufacturer’s instructions. Results of NE, IL-8, and TNF-R2 assays were interpreted using standard curves appropriate for individual kit protocols. Galactomannan was measured using ratios of optical densities (OD). A positive result was based on a GM optical density index (ODI) of ≥0.5.

An optimization step was undertaken due to homogenization of our respiratory samples with DTT. All inflammatory mediator kits underwent preparation of standards with and without DTT for comparison. This validation step did not demonstrate any significant variations in the concentration of standards for biomarker assay kits.

### Statistical Analysis

We present summary statistics as median (range) for continuous data and as frequency (%) for categorical data. The comparison of baseline characteristics across both groups of fungal culture was performed using Mann-Whitney U test for continuous data and Chi-squared tests for categorical data. Analysis of normally distributed data including the association of lung function with age was performed using the independent samples t-test. Statistical significance was at the 0.05 level. The association between clinical characteristics across both categories of fungal culture was investigated with the use of a binomial regression model and reported as p-values (Wald test) and odds ratios (OR). The following independent variables; age, antibiotic use, bronchiectasis, lung function and immunological markers on serology were used for this logistic regression model to determine the association with fungal culture positivity (dependent variable).

## Results

### Cohort Characteristics

**Figure 1:**
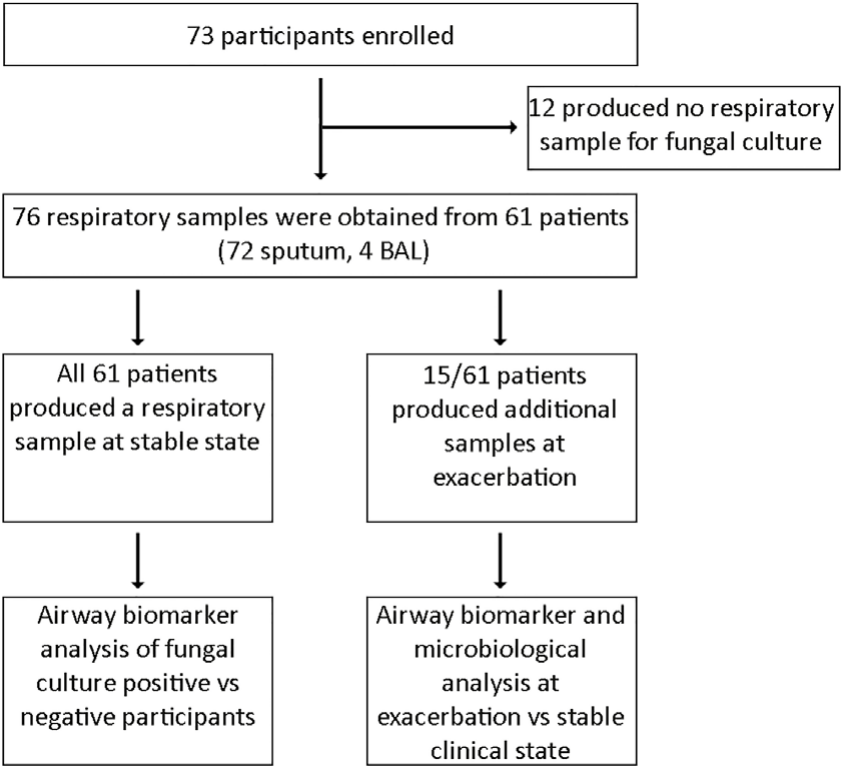
Consort diagram.

Sixty-one of the 73 patients enrolled provided respiratory specimens and were subsequently eligible for inclusion in our study. Twelve patients were excluded as no respiratory sample for fungal culture was obtained (**Figure 1**). A total of 76 respiratory specimens were processed for fungal culture, which comprised of 72 sputum and four BAL samples. Median age at recruitment was 17 years (range 6 months–59 years) with 29 male participants and a median percent predicted FEV_1_ of 77% (range 21–120%). All 61 participants provided a sputum sample during the stable state, of whom 43 patients also produced respiratory samples suitable for biomarker analysis. Of the respiratory samples that underwent biomarker analysis (n = 43), only three samples were derived from BAL. Fifteen patients provided a further respiratory sample during clinical exacerbation to enable comparison with their paired stable state sample.

### Clinical Characteristics in Relation to Fungal Culture

Thirty-one patients (49%) cultured filamentous fungi from their sputum and/or BAL during a clinically stable state. Concomitant growth of *A. fumigatus* and yeast was the commonest (60%) within the fungal culture positive group, followed by *A. fumigatus* alone (40%). No other filamentous fungi were detected.

The median age within the fungal culture positive cohort was 15 years (range 7–53 years). No child under age seven years was observed to be fungal culture positive. Patient demographics, clinical data and cultured pathogens are presented in **Table 1**. No significant differences were observed between the distribution of age, BMI, genetics, indoor mold exposure (as defined by the presence of self-reported household mold) and pancreatic status between the two groups.

**Table 1:** Clinical characteristics in CF with and without positive sputum for filamentous fungi at stable state.

Variable	Fungal culture positive		Fungal culture negative		P value
	Total N		Total N		
Male n (%)	30	13 (43%)	31	16 (52%)	0.517
Age (years)	30	15 (7–53)	31	16 (0.5–59)	0.919
DF508 homozygous n (%)	30	13 (43%)	31	14 (45%)	0.886
Pancreatic insufficient n (%)	30	26 (87%)	31	28 (90%)	0.654
BMI (kg/m^2^)	30	20.8 (13.5–30.1)	31	19.5 (15.3–27.6)	0.573
Indoor mold exposure n (%)	29	12 (40%)	30	9 (29%)	0.659
Ivacaftor n (%)	30	1 (3%)	31	3 (10%)	–
Prophylactic antibiotics n (%)	29	16 (55%)	30	17 (56%)	0.993
Nebulized antibiotics n (%)	29	14 (48%)	30	14 (47%)	0.992
Inhaled steroids n (%)	28	19 (68%)	31	10 (32%)	**0.008**
Oral steroids n (%)	29	3 (10%)	31	1 (3%)	–
3 monthly ivab n (%)	29	11 (38%)	30	5 (17%)	0.185
SA colonization n (%)	29	6 (21%)	30	4 (13%)	0.753
PA colonization n (%)	29	8 (28%)	30	9 (30%)	0.979
Previous NTM n (%)	29	12 (41%)	30	3 (10%)	**0.022**
Bronchiectasis on CT n (%)	17	13 (76%)	11	7 (64%)	0.225
ABPA n (%)	29	4 (14%)	30	6 (20%)	–
Total IgE (KU/L)	29	156 (5–4,433)	29	76 (3–4,273)	0.260
Asp. IgE (KUA/L)	27	0.33 (0–41)	30	0.05 (0–37.8)	0.378
Asp. IgG (mg/L)	29	58 (15–185)	28	52 (6.8–181)	0.139
FEV_1_ predicted (%)	30	72 (21–122)	28	80 (24–131)	0.920
FEV_1_/FVC (%)	30	70 (32–97)	28	72 (41–92)	0.767
LCI	9	11.6 (7.75–20.72)	11	7.84 (6.41–13.85)	0.972
Sputum lymphocyte count (%)	13	0 (0–0.5)	11	0 (0–5.75)	0.289
Sputum macrophage count (%)	13	2.75 (0–32)	11	2 (0.25–28.25)	0.511
Sputum eosinophil count (%)	13	0.1 (0–2.75)	11	0.25 (0–14.5)	0.631
Sputum neutrophil count (%)	13	94.88 (67.75–99.5)	11	92.75 (10.25–98.5)	0.887

IVAB, Intravenous antibiotics; ICS, inhaled corticosteroid; SA, Staphylococcal aureus; PA, Pseudomonas aeruginosa, NTM, non-tuberculous mycobacteria; IgE, Immunoglobulin E; Asp IgE, Aspergillus specific IgE; Asp IgG, Aspergillus specific immunoglobulin G; FEV1, Forced expiratory volume in one second; FVC, Forced vital capacity; LCI, Lung clearance index. Colonization is defined as >50% samples positive over 12-month period.

Categorical data is presented as frequency (n) and analyzed using Chi-squared test. Continuous data is presented as median and range. Non-parametric data was analyzed as comparison of medians across groups using the Mann-Whitney U test. Statistical significance was defined as P value < 0.05. Bold values signify statistical significance at P < 0.05.

We observed significantly higher rates of non-tuberculous mycobacteria (NTM) isolates within the fungal culture positive sub-group (p = 0.022). This finding was not noted for co-colonization with other bacteria such as *Staphylococcus aureus* or *Pseudomonas aeruginosa*.

We report greater use of inhaled corticosteroids within the FC positive cohort compared to the FC negative cohort (p = 0.008).

A binomial regression analysis was carried out to predict whether fungal culture positivity is associated with clinical characteristics including age, sex, mold exposure, routine microbiology, medication use, and lung function. Fungal culture positivity was 14 times more likely in the presence of NTM (p= 0.007) and five times more likely with indoor mold exposure (p = 0.047). Although not statistically significant, the use of three-monthly intravenous antibiotic was four times more likely to be associated with fungal culture positivity. Significant associations for the predictor of fungal culture positivity were not demonstrated for other independent variables highlighted in **Table 2**. Missing values within the binomial regression model were accounted for by use of median values (N <4 for independent variables including prophylactic, nebulized and intravenous antibiotic use, lung function, microbiology and serological markers). Due to multiple missing values for the independent variables LCI and bronchiectasis, a regression model could not be utilized without introducing significant bias.

**Table 2:** Binomial regression analysis of clinical characteristics in relation to fungal culture positivity.

Variable	P value	Exp(B)/OR 95% CI
Age (years) Sex	0.542 0.220	1.024 (0.948–1.107) 0.354 (0.067–1.860)
Pancreatic insufficiency	0.469	0.167 (0.396–7.486)
Indoor mold exposure	**0.047**	5.217 (1.025–26.545)
Prophylactic antibiotic use	0.546	0.635 (0.146–2.773)
Nebulized antibiotic use	0.801	0.822 (0.180–3.750)
3 monthly ivab exposure	0.108	4.761 (0.712–31.836)
FEV_1_ predicted (%)	0.351	0.985 (0.953–1.017)
S. aureus	0.514	1.845 (0.293–11.601)
P. aeruginosa	0.869	1.175 (0.170–7.984)
NTM	**0.007**	14.263 (2.064–98.540)
Total IgE (KU/L)	0.264	1.001 (0.999–1.002)
Asp. IgE (KUA/L)	0.307	0.930 (0.810–1.069)
Asp. IgG (mg/L)	0.401	1.007 (0.990–1.025)

IVAB, Intravenous antibiotics; Forced expiratory volume in one second; IgE, Immunoglobulin E; Asp IgE, Aspergillus specific IgE; Asp IgG, Aspergillus specific immunoglobulin G. P values are derived from the Wald test with statistical significance defined as <0.05.

**Figure 2:**
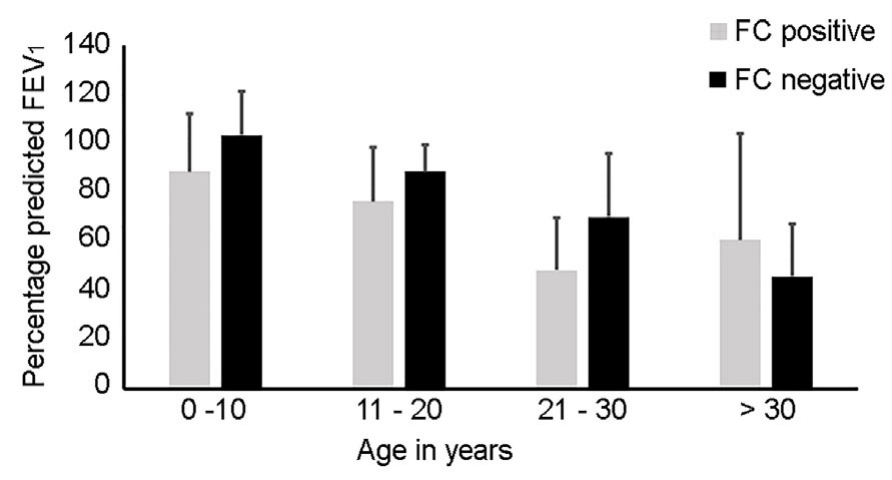
Histogram representing differing age groups in relation to fungal culture (FC) positivity and percentage predicted Forced Expiratory Volume in 1 second (FEV_1_). The lung function data is presented as the standard error of mean per age category. The mean FEV_1_ is lower within all age groups within the FC positive group with the exception of the over 30-year sub-group; 0–10 years (FC positive mean 89%, 95% CI 64–113; FC negative mean 103%, 95% CI 84–122, p = 0.25), 11–20 years (FC positive mean 76%, 95% CI 63–90; FC negative mean 89%, 95% CI 77–100, p = 0.225), 21–30 years (FC positive mean 48%, 95% CI 29–68; FC negative mean 70%, 95% CI 48–92, p = 0.015) and >30 years (FC positive mean 61%, 95% CI 8–129; FC negative mean 46%, CI 28–64, p = 0.434).

The trend in lung function measures in relation to age and fungal culture positivity is shown in **Figure 2**. The mean FEV1 is lower within the fungal culture positive cohort at all ages with the exception of the over 30-year group. A significantly lower FEV1 was noted within the 21–30-year old fungal-culture positive group (p = 0.015). Although not statistically significant, we noted a higher FEV1 within the over 30-year old fungal-culture positive subgroup.

Although not significant, we also note an increased LCI within the fungal culture positive cohort at all ages in comparison to the fungal culture negative cohort.

Similar rates of ABPA were noted between the two cohorts with 13% in the fungal culture positive group (n = 4) and 19% in the fungal culture negative group (n = 6).

### Biomarker Analysis

#### Neutrophil Elastase

**Figure 3:**
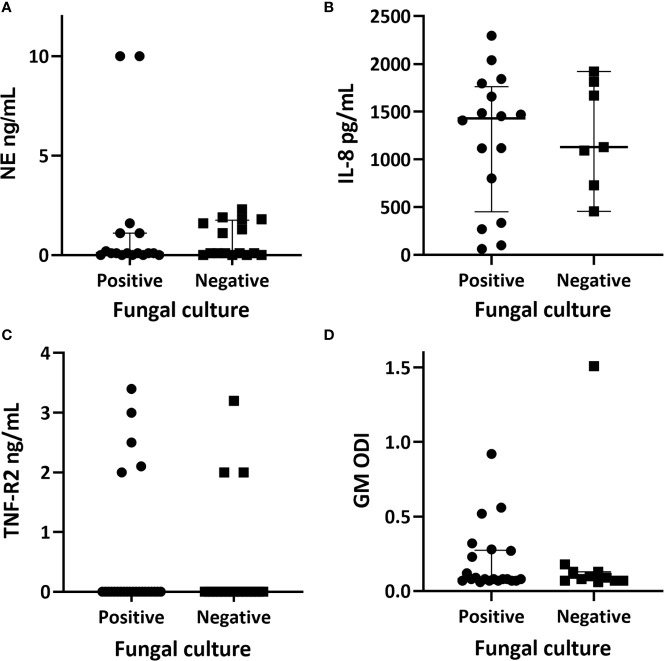
Quantitative airway biomarker measurements in relation to fungal culture positivity. **(A)** neutrophil elastase with the fungal culture positive group having a median value of 0.1 ng/ml (range 0–10) and the culture negative group had a median of 0.1 (range 0–2.3); p = 0.107. **(B)** interleukin-8 with a median of 1,430.45 pg/ml (range 62.9–2,296.2) within the fungal culture positive group and a median of 1,128.3 (range 455.2–1,921.7) within the fungal culture negative group; p = 0.871. **(C)** tumour necrosis factor receptor 2 (TNF-R2) with a median of <2 ng/ml within both subgroups. **(D)** galactomannan values represented as optical density index (ODI) with the fungal culture group having a median of 0.09 (range 0.06–0.92) and the culture negative group with a median of 0.08 (range 0.066–1.44); p = 0.143. Individual data, median and interquartile range are shown. Comparison of medians between groups were undertaken using the Mann-Whitney U test for non-parametric data.

All samples were processed for NE however only 33/43 (77%) yielded a result as ten readings were unavailable due to insufficient sample. Twenty four of the 33 samples (73%) had a detectable NE value of ≥0.1 ng/ml (reference range 0.16–10 ng/ml). The remaining samples had a value of <0.1 ng/ml, which fell outside of the detectable range on the standard curve. There was no significant difference in NE between the fungal culture positive group (median value 0.1 ng/ml, range 0.1–10) and the fungal culture negative group (median 0.1 ng/ml, range 0.1–2.3, p = 0.107) (**Figure 3A**).

Two patients had high NE levels (> 10 ng/ml), both of whom were fungal culture positive. The first patient had serological evidence of ABPA with a serum IgE of 4433 kU/L, *Aspergillus* specific IgE of 25.1 kUA/L, and an *Aspergillus* specific IgG of 130 mg/L. This patient was receiving three-monthly intravenous antibiotics and nebulized antibiotics as part of M*ycobacterium abscessus* eradication regime. The second patient had mixed growth of *A. fumigatus* and *Haemophilus influenzae* on sputum culture, no pre-existing history of fungal lung disease, normal serological markers, and was not receiving regular intravenous antibiotics.

#### Interleukin-8

Sufficient sputum was available for 23 samples to be tested for IL-8 (reference range 15.6–1,000 pg/ml). All assays yielded a detectable result of >62 pg/ml (reference range 15.6–1,000 pg/ml) (**Figure 3B**). Sixteen of the 23 respiratory samples (70%) tested were from fungal culture positive patients. The comparative median and ranges of IL-8 between the fungal culture positive and negative groups are reported in **Figure 3**. There was no significant difference in IL-8 between the fungal culture positive and negative sub-groups (p = 0.871).

#### Tumor Necrosis Factor Receptor 2

All respiratory samples were tested for TNF-R2 (reference range 2–142 ng/ml). Eight patients (19%) had a detectable level of ≥2 ng/ml (**Figure 3C**). Five of the eight were fungal culture positive. Both groups had a median TNF-R2 below the detectable range of the assay.

Participants with detectable TNF-R2 levels were not noted to have any discernible patterns to lung function impairment, lung clearance index, serological markers, intravenous antibiotic use, or radiological evidence of bronchiectasis on CT.

#### Galactomannan

There was enough sputum for galactomannan testing available for 32 patients. Only four had a positive result with ≥0.5 ODI, three of which fell into the fungal culture positive group (**Figure 3B**).

### Paired Sputum Analysis

**Figure 4:**
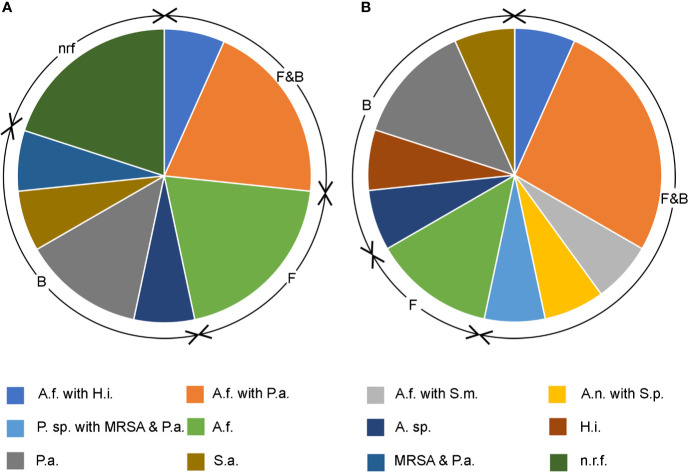
Pie charts showing bacterial and filamentous fungal isolates cultured from respiratory samples during **(A)** stable state and **(B)** acute exacerbation. F&B; concomitant fungal and bacterial growth, F; fungal only, B; bacterial only, nrf; normal respiratory flora. Fungi cultured: A.f. *Aspergillus fumigatus*, A.n. *Aspergillus niger*, P. sp. *Penicillium* sp. Bacteria cultured: A. sp. *Achromobacter* sp., H.i. *Haemophilus influenzae*, P.a. *Pseudomonas aeruginosa*, S.a. *Staphylococcus aureus*, MRSA methicillin resistant *S. aureus*, S.m. *Strenotrophomonas maltophilia*, S.p. *Streptococcus pneumoniae*.

Fifteen patients provided paired sputum samples obtained at routine exacerbation and stable state visits. Ten (67%) were positive for filamentous fungi during an exacerbation state and seven (50%) during the stable state. All but two participants’ samples were culture positive for *A. fumigatus*. The other two samples were positive for either *Penicillium species* or *Aspergillus niger*, both during the acute exacerbation state. **Figure 4** demonstrates a higher proportion of concomitant bacterial growth during the exacerbation state. Ninety-three percent had mixed bacterial and fungal growth during acute exacerbation in comparison to 60% during the stable state. Seven patients had the same bacterial species detected during exacerbation and stable states and represented chronic colonization. No participants during exacerbation were fungal culture negative and exhibiting only normal respiratory flora, compared to 20% (n = 3) at stable state.

Due to a lack of sufficient sputum volume, only three paired samples were available for biomarker comparison between pulmonary exacerbation and stable disease. NE (1.2 ng/ml exacerbation vs 0.05 ng/ml during stable state) and GM (0.5 OD exacerbation vs 0.14 OD stable) were higher during pulmonary exacerbation (p < 0.001), with a trend towards higher IL-8 levels during the exacerbation state (1,842.4 pg/ml exacerbation vs 1,335.3 pg/ml during stable).

## Discussion

Our study is the first to describe CF airway biomarkers in relation to fungal culture positivity. We report the relationship between airway fungal culture positivity, airway biomarkers and clinical, radiological and microbiological characteristics in children and adults with CF.

The clinical relevance of fungal airway colonization and its impact on lung disease progression in CF is unclear. In keeping with previous reports, we found that *A. fumigatus* was the most frequently isolated fungal species (Pihet et al., 2009). Colonization with *A. fumigatus* has been associated with more advanced CF lung disease (Ramsey et al., 2014), lower lung function (Amin, 2010; Saunders et al., 2016) and greater risk of pulmonary exacerbations requiring hospitalization (Amin, 2010). In our study, the fungal culture positive group, with the exception of the over 30-year old sub-group, had a lower mean FEV1 across all younger age groups. Our findings of a higher FEV1 within the FC positive subgroup for the over 30-year age group, may be a reflection of participants surviving with milder CF lung disease.

LCI derived from multiple breath washout is a more sensitive marker of lung disease progression compared to spirometry, allowing non-invasive monitoring of small airways disease. A study by Walicka-Serzysko et al. demonstrated a higher LCI in CF patients with *A. fumigatus* (Walicka-Serzysko et al., 2019). We also found higher LCI’s in fungal culture positive patient but this did not reach statistical significance.

The primary focus of previous studies have included exploring the association between *A. fumigatus* and clinical outcomes, there is a paucity of information exploring fungal airway colonization and its impact on markers of airway inflammation. It is well established that the CF airway milieu contains higher levels of pro-inflammatory cytokines in comparison to healthy controls (Sagel, 2009). Evidence for pulmonary inflammation in surveillance BAL such as raised NE and IL-8 in the CF airways is present, even in the absence of clinically apparent lung disease (Armstrong et al., 2005; Frayman et al., 2017; Khan et al., 2019). Sly et al. have reported a link between high NE levels within the CF airways and the subsequent development of bronchiectasis (Sly et al., 2009). Whether fungal colonization exacerbates underlying airway inflammation, resulting in higher levels of airway biomarkers and therefore further driving lung disease progression requires further study. Exploring this relationship is complicated by the difficulty in separating the effect of bacteria and fungi present in respiratory cultures.

In our study, only one-third of patients were fungal culture positive without the presence of concomitant pathogenic bacteria, making the direct attribution of inflammation to a positive fungal culture problematic. We found no significant difference between NE and IL-8 between the fungal culture positive and negative groups. Two patients with very high levels of NE were fungal culture positive but these low numbers preclude any firm conclusion. Studies have shown improvements in NE and IL-8 levels following treatment of an exacerbation (Ordonez et al., 2003; Colombo et al., 2005). Evidence suggests NE activity is higher during exacerbation and is responsive to antibiotics (Chalmers et al., 2017). Whilst we acknowledge our small number of paired patient samples due to difficulty in obtaining sufficient sputum volumes, we report higher levels of NE and IL-8 during pulmonary exacerbation compared to the stable state.

Galactomannan may be a useful screening test for invasive pulmonary aspergillosis (Kimura et al., 2009; Talento et al., 2017). The diagnostic usefulness of galactomannan assays in patients with non-invasive fungal lung disease in CF, such as ABPA and chronic pulmonary aspergillosis remains uncertain (Nguyen et al., 2007; Kitasato et al., 2009; Fayemiwo et al., 2017). We found that only four patients (13%) within our cohort had a positive result for galactomannan. There were no significant difference in the absolute values of galactomannan between the fungal culture positive and negative groups. Contrary to our study findings, galactomannan has been reported as a sensitive method of detecting *Aspergillus* in sputum in comparison to fungal cultures (Baxter et al., 2013a), even in the absence of invasive disease. Baxter et al. used sputum samples homogenized with Sputasol (commercial product containing DTT) and sonication. In comparison we used homogenized sputum supernatants without sonication, which may have decreased the overall yield.

There is emerging evidence implicating TNF-R2 as a possible airway biomarker in fungal associated asthma. Ghebre et al. noted significantly higher TNF-R2 levels in adults with asthma who were fungal culture positive (Ghebre et al., 2017). Our study is the first to analyze TNF-R2 as a potential biomarker of inflammation in adults and children with cystic fibrosis. Overall, we detected TNF-R2 in only 19% of our patients. Within the limitations of our modest number of patients with detectable TNF-R2 levels, we found no association with adverse clinical outcomes. Whilst Ghebre et al. concluded that TNF-R2 may diminish the inflammatory response to *A. fumigatus*, the exact mechanism for this and its clinical implications in CF is unknown. Interestingly, Ghebre et al. concluded that TNF-R2 appears to be a strong discriminator of exacerbation in children and adults with asthma (Ghebre et al., 2019). Further studies in CF are required to determine if a similar relationship exists between fungal culture positivity, exacerbation states and higher TNF-R2 levels.

We found that the fungal culture positive subgroup had greater previous and/or current positive sputum isolates for non-tuberculous mycobacteria. This is in keeping with previous literature where it has been proposed that *Mycobacterium* superinfection can occur alongside aspergillosis (Cui et al., 2013). The proposed theories include a synergistic relationship which enhances their resistance to environmental change such as use of broad-spectrum antibiotics (Kerr, 1994; Cui et al., 2013).

Within our CF cohort, we report higher rates of fungal culture positivity with indoor mold exposure. Evidence suggests a link between indoor fungal contamination and ABPA. Reports linking airway fungal colonization and mold exposure in CF are scarce (Rocchi et al., 2015). Fairs et al. reported environmental mold exposure may predispose asthmatics to airway colonization (Fairs et al., 2013) and whether this also occurs in CF patients is unclear and requires further study.

Although not statistically significant we observed a higher rate of bronchiectasis in the fungal culture positive cohort with 76% having radiological evidence of this on CT chest, in comparison to 64% of the culture negative cohort. Similar associations have been reported between *A. fumigatus* and higher radiological scores with more significant bronchiectasis (McMahon et al., 2012).

Whilst over 35% of our fungal culture positive cohort received three-monthly intravenous antibiotics, only 16% of fungal culture negative group were exposed to regular antibiotics. Several studies have shown an association between the use of broad-spectrum antimicrobials and *A. fumigatus* airway colonization (Burns et al., 1998; Hodson et al., 2002). The use of regular intravenous antibiotics are typically reserved for patients with more severe lung disease and/or bacterial colonization. The concept of regular exposure to broad spectrum antimicrobials reducing competition for surfaces and limited nutrients, therefore promoting fungal adhesion, growth and colonization has therefore been proposed (Oever and Netea, 2014). However, a study by Baxter et al. reported a significant decline in the bioburden of *Aspergillus* when they compared pre- and post-antibiotic sputum samples from 26 adult CF patients (Baxter et al., 2013b). Therefore, the use of antimicrobials for well-established CF pathogens and its impact on *A. fumigatus* remains poorly understood.

We report a significantly higher rate of inhaled corticosteroid use within the FC positive cohort. This is a well-established finding in existing literature whereby the use of inhaled and/or systemic corticosteroids are known to promote *A. fumigatus* growth (Noni et al., 2015).

Our study limitations include a modest sample size, in particular for the paired sample analysis. The presence of significant numbers of non-sputum producing children in our study cohort means that we were unable to obtain sufficient volumes to complete ELISA biomarker and sputum cell differential analysis for all patients. We included a combination of sputum and BAL samples to enhance our sample size and to allow inclusion of non-sputum producing children within our study cohort. In order to minimize bias resulting from salivary contamination of respiratory samples, we included BAL samples when clinically indicated and where we were unable to obtain spontaneous or induced sputum samples.

Secondly, published studies report a wide range of prevalence in the detection of filamentous fungi (Nagano et al., 2010; Pashley et al., 2012) due to sampling techniques and culture conditions, it is likely that the true prevalence of fungi in CF is underestimated. To overcome this we used a modified fungal culture approach, which has been shown to be a more sensitive for the detection of filamentous fungi (Pashley et al., 2012). Whilst it is possible that our approach is detecting low levels of fungi that are not truly colonizing the lower airways, the association between higher culture rates and worse lung function in both this and the adult asthma studies (Fairs et al., 2010; Agbetile et al., 2012) that have utilized this more sensitive culture method would suggest the detection may be clinically relevant. An alternative approach to culture may be to utilize a molecular technique such as quantitative polymerase chain reaction (qPCR), which is commercially available for *A. fumigatus* and a limited number of other *Aspergillus* species (Guegan et al., 2018). Both modified fungal culture and *Aspergillus* quantitative PCR have been shown to be more sensitive than the NHS routine approach (Public Health England, 2019) for identifying *A. fumigatus* from respiratory secretions (Fraczek et al., 2014). It should be noted, however, that the current commercial qPCR assays, whilst based on quantitative technology, are used for determining the presence or absence of a fungus only, and will amplify DNA from dead as well as live fungal material.

Our study reports on the short-term clinical outcomes associated with FC positivity in CF given the cross-sectional methodology used. Whilst the long-term outcomes of ABPA and serological sensitization are well-described with evidence of lung function decline (Kraemer et al., 2006), increased need for intravenous antibiotics (Peetermans et al., 2014) and bronchiectasis (Agarwal et al., 2010), literature on the outcomes of *Aspergillus* colonization and non-invasive infection is scarce. It is clear that further prospective longitudinal studies are required to describe the clinical outcomes of *A. fumigatus* colonization and non-invasive infection.

Finally, statistical analysis for LCI and bronchiectasis were excluded from the binomial regression model due to the risk of bias from missing data. A limited number of patients had LCI results available as this is not routine practice in our adult CF cohort and is carried out as part of the annual comprehensive review process in children. However, not all children are capable of completing this procedure. We also note a limited number of patients underwent a CT chest as this was only carried out as part of routine care when indicated. In addition, our results obtained for NTM and mold exposure have high confidence intervals despite clinical significance, reflecting the highly variant estimates derived for these parameters.

In conclusion, we found no difference between the airway biomarker profile between sputum fungal culture positive and negative patients with CF. The role of galactomannan and TNFR2 as more fungal specific airway biomarkers in CF remains uncertain. Fungal culture positivity is associated with a lower FEV1 in patients less than 30 years old, a lower LCI, higher rates of NTM positivity, bronchiectasis and intravenous antibiotic exposure. Larger trials using a broader range of inflammatory markers in conjunction with fungal culture and quantitative PCR measures are needed.

## Data Availability Statement

The original contributions presented in the study are included in the article/supplementary materials. Further inquiries can be directed to the corresponding authors.

## Ethics Statement

The studies involving human participants were reviewed and approved by Leicester longitudinal study of respiratory infections and microbiomics in CF by the East Midlands Research Ethics Committee, reference number 12/WM/0285. Written informed consent to participate in this study was provided by the participants’ legal guardian/next of kin.

## Author Contributions

DP, CP, and EG co-designed the study. DP was responsible for analyzing the data and writing the manuscript. CP supervised the laboratory analyses (modified culture and ELISA) and EG the clinical aspects of the study. DP was responsible for patient recruitment, the design and custodianship of the clinical database, and processing clinical samples. DP and KD undertook the ELISA analysis. All authors contributed to the article and approved the submitted version.

## Funding

Funding from Gilead Sciences Grant Program (grant no. 02261) and University Hospitals of Leicester charitable funds was awarded for this study alongside our separate study termed “Changes in the Cystic Fibrosis Airway Mycobiome Over Time and its Link with Bacterial Species Rarefication”.

## Conflict of Interest

DP reports grants from Gilead Sciences Grant Program, outside the submitted work. This grant was awarded for a separate ongoing study termed: Changes in the Cystic Fibrosis Airway Mycobiome Over Time and Its Link with Bacterial Species Rarefication. CP has received research support and honorariums for advisory board from Pulmocide who are developing a new antifungal agent. EG reports from Consultancy work for Boehringer Ingelheim November 2016, from Consultancy work for Anaxsys July 2018, grants from Circassia research grant, grants from Gilead sciences research grant, grants from Chiesi Limited, from Medimmune research collaboration, and outside the submitted work: 1. Consultancy work for Boehringer Ingelheim November 2016. Money, paid to the institution (The University of Leicester); 2. Consultancy work for Anaxsys July 2018, money paid to the institution (The University of Leicester); 3. Circassia research grant, investigator-led research grant; 4. Gilead sciences research grant, investigator-led research grant; and 5. Medimmune research collaboration, research collaboration. 

The remaining author declares that the research was conducted in the absence of any commercial or financial relationships that could be construed as a potential conflict of interest.
